# Measuring Cortisol in the Classroom with School-Aged Children—A Systematic Review and Recommendations

**DOI:** 10.3390/ijerph15051025

**Published:** 2018-05-18

**Authors:** Mirena Dimolareva, Nancy R. Gee, Karen Pfeffer, Laëtitia Maréchal, Kyla Pennington, Kerstin Meints

**Affiliations:** 1 School of Psychology, University of Lincoln, Sarah Swift Building, 8 Brayford Wharf, Lincoln LN5 7AT, UK; mDimolareva@lincoln.ac.uk (M.D.); KPfeffer@lincoln.ac.uk (K.P.); LMarechal@lincoln.ac.uk (L.M.); KPennington@lincoln.ac.uk (K.P.); 2 Department of Psychology, State University of New York, Fredonia, NY 14063, USA; Nancy.gee@fredonia.edu; 3 WALTHAM™ Centre for Pet Nutrition, Waltham-on-the-Wolds, Melton Mowbray, Leicestershire LE14 4RT, UK

**Keywords:** cortisol, collection, school, child, special educational needs, typically developing children

## Abstract

The collection of salivary cortisol has been chosen as one of the least intrusive, easiest to collect, analyze, and store methods of obtaining information on physiological changes. It is, however, not clear what the best practice is when collecting salivary cortisol from children within the school setting. The aim of this systematic review is to evaluate the feasibility of cortisol collection in schools for future research and to make recommendations for best practice. The review included 25 peer-reviewed articles from seven databases. The hypotheses of the included studies vary, but they all use cortisol as a diurnal, baseline, or acute measure, or to measure the effect of an intervention. Two methods of salivary cortisol collection were preferred by most of the research, i.e., passive drool or cotton Salivettes. The review has concluded that cortisol is a physiological marker that can be successfully measured in school-based research. However, there are discrepancies across studies when evaluating the collection guidelines, protocols, and instructions to participants as well as transparency of the success rate of obtaining all samples. Recommendations are made for future research to address and avoid such discrepancies and improve cross-study comparisons by implementing standard protocol guidelines.

## 1. Introduction

How feasible is cortisol collection for large-scale cohort studies in schools? Research in schools provides the advantage of investigating children in their daily school environment (increasing ecological validity), while also allowing researchers to measure a larger group of similar-aged children in similar circumstances and learning conditions (which likely reduces measurement error). This has also enabled researchers to compare results of typically developing children with children with learning difficulties such as Autism Spectrum Disorder (ASD) and Attention Deficit Hyperactivity Disorder (ADHD) [1,2,3].

It is desirable to collect a variety of measurements to gain a more complete understanding of children’s behavior. These should include both indirect (e.g., questionnaires) and direct (e.g., objective physiological) measures. However, when working with children, researchers may avoid collecting physiological data due to the complexities of this task. For example, these types of measurements can be uncomfortable for the children (e.g., a tightening blood pressure cuff, or a needle stick for a blood sample), which may cause them to withdraw from the study, creating a variety of problems associated with missing data.

Interventions in schools, for example animal-assisted interventions (AAI) [4,5], or yoga and mindfulness interventions, have successfully measured cortisol to see how these interventions have affected children and their stress levels [6,7]. Some research has used salivary cortisol to investigate how aroused or relaxed typically developing children [8], children with clinical disorders such as ADHD [1,2], and Obsessive-Compulsive Disorder (OCD) [9], were feeling at a given time. Other studies have established the natural decline of cortisol through the day within the classroom [10]. Thus, salivary cortisol has proven to be a feasible physiological measure to collect. As a result, it has provided an insight into the physiological changes that occur in children as it indicates arousal which is used as a measure of stress.

Cortisol is a major glucocorticoid hormone produced in the adrenal cortex, known to increase in stressful situations. Cortisol has a diurnal pattern, it tends to show an increase in the morning followed by a decline throughout the day [11]. This differs between individuals and is influenced by many factors such as acute life events [12], diagnosis of disorders such as ADHD [13], socio-economic status [14,15], and classroom environment [16]. In general, depending on the research question, cortisol can be measured as a diurnal curve, Cortisol Awakening Response (CAR), or as baseline or acute stress measures (for more detailed information see [17]).

There are three different sampling methods used to measure cortisol levels in the body, and these include the following: blood [18], urine [19], or saliva [1,2,20]. The least intrusive of these methods, and the easiest to collect is salivary cortisol collection [21]. For this reason, it is often the method of choice by researchers, especially those working with children. Different approaches to the collection of salivary cortisol have been established, with the most popular among them being passive drool (where participants drool into a tube) or using cotton swabs called Salivettes (participants keep the cotton swab in their mouth for 1–2 min, until saturated with saliva). These methods appear to be used with roughly equal frequency. While some research findings suggest the use of Salivettes as a preferable method of establishing free cortisol [22], others have concluded that passive drool or the use of cellulose sponge sorbettes provides more stable readings of saliva [23]. In addition, an established advantage of passive drool is that it collects the whole saliva (from different areas of the mouth) rather than saliva from a particular area (e.g., where the sorbette or Salivette has been left to be saturated with saliva) of the mouth and therefore provides the purest sample [24,25]. Additionally, the percentage of cortisol recovered from such tools can vary [26]. This in turn prompts questions with regards to the comparability of results across studies, as it is unclear whether the two methods produce similar results. An alternative method for young children similar to sorbettes can be taking a swab (e.g., SalivaBio’s Children’s Swab consisting of a durable polymer), which is usable with infants from 6 months on and is especially useful when infants or children cannot give enough saliva with passive drool (e.g., [27]).

Furthermore, obtaining saliva samples is not always easy as successful collection depends on the implementation of strict protocols by parents at home [28], as well as the child’s compliance with the procedure [29]. This can be difficult to control in instances where parents help children to provide the samples at home without the moment-to-moment oversight of a researcher. However, some researchers have encouraged the following of protocol by providing parents with journals for recording exact time of collection [6,30]. Parents have also been given advice to set alarms to remind them of the collection times [31] and some researchers have even arranged to model the procedure and give training prior to the day of collection [3,30]. Despite all efforts, there are frequently children who do not provide all the saliva samples for analysis [16]. However, despite the missing data, cortisol is a measure which researchers use for different research purposes because of its flexibility and relative stability.

To assess the feasibility of cortisol collection for large-scale cohort studies in schools, this review aims to provide an overview of different methods of cortisol collection attained from children in classroom settings, including typically developing children and children with special educational needs. Information on the protocol and method of collection, number, and time of samples collected, as well as the storage prior to analysis, will be discussed. As the intent is to consider implications for research conducted within the classroom setting, any studies using samples collected solely at home or outside the classroom were not included. This approach eliminates the factor of parent non-compliance. Concentrating on the samples taken in the classroom ensures a more accurate representation of limitations in this applied setting. This will in turn allow for more accurate recommendations and guidelines to be suggested which will help create a gold standard protocol for future research. Such guidelines are particularly important as cortisol is a useful, nonintrusive, and fairly easy to collect biological marker of arousal, HPA-axis function, and stress reactivity. Collecting this type of direct physiological measurement has the potential to enhance information gained from indirect measures which rely, for example, on participant self-reports or test questions, which may not always be answered as truthfully or accurately as possible.

## 2. Materials and Methods

Only cortisol measurements taken at school with school-age children were included. These measures included the following: diurnal cortisol changes occurring through the day, cortisol measured in relation to an intervention, baseline cortisol, and acute cortisol. As mentioned above, with this protocol, we eliminated the factor of parent non-compliance, which is a factor in studies measuring cortisol levels throughout the day. Collection methods have been evaluated. This included the number of saliva samples collected, the protocol followed, the method of collection, and the storage of samples. The scope of this review did not extend to other interventions and measures used alongside the cortisol collection.

Eligibility criteria and search methods were defined before the search took place. The PRISMA guidelines and PRISMA checklist [32] were followed. Articles were selected according to the following criteria: (i) all articles were written in English, (ii) articles were published in peer-reviewed journals, (iii) all or some of the cortisol samples were collected in a classroom setting when children were in full-time education (therefore excluding studies with pre-school/nursery-aged children), and (iv) case studies and literature reviews were not included. Articles from seven databases were included: Academic Search Complete, MEDLINE, PsychArticles, PsychInfo, Science Direct, Web of Science, and Scopus. The keywords used were cortisol related: “salivary cortisol” “Hypothalamic-Pituitary-Adrenal axis”, “HPA”, and were combined with one of the following to obtain typically developing children and children with any special educational needs: “school children”, “classroom”, “education”, “ASD”, and “ADHD”. The relevant research articles were reviewed by two of the authors.

## 3. Results

**Figure 1:**
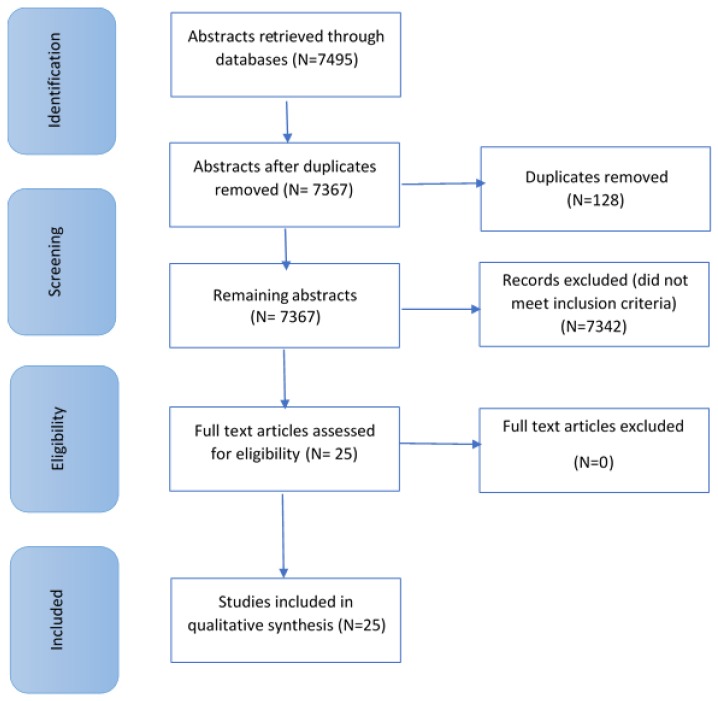
Flow chart adapted from PRISMA, showing the selection process and criteria of papers included in this review.

From this search, 7495 articles were returned. Articles not fitting the eligibility criteria were excluded. Articles were excluded due to cortisol samples being collected at home, in pre-school/nursery settings, during non-school days, and in-patient clinics. Twenty-five articles remained (see Figure 1 below).

These articles were categorised using the Oxford Centre for Evidence-Based Medicine 2011 Levels of Evidence and articles fall into two categories: Non-randomized controlled cohort/follow-up study and randomized trial or observational study with dramatic effect (see Table 1).

In order to discuss the relevant research, the 25 selected studies were divided into the following subsections depending on what they were using the cortisol measure for: (1) measuring diurnal cortisol changes occurring through the day, (2) measuring whether an intervention has an effect on participant cortisol levels, (3) cortisol as a baseline/comparison measure, and (4) measuring stress responses (acute measure). Results are depicted in overview in Table 2 below.

### 3.1. Diurnal Measure of Cortisol

Some studies returned in the search measured the daily secretion of cortisol. This included the Cortisol Awakening Response (CAR) measured upon awakening and 30 min after, as well as samples taken throughout the day which show an initial steep decline followed by a more gradual decline [30,31,40]. For the purposes of this review, only the samples taken during the day at school will be discussed, as the aim of the review is to assess the feasibility of cortisol collection and to discuss the protocol for collection, method, and success rate for saliva samples collected specifically within the school environment.

Two of the studies used a cohort of typically developing children. Firstly, the study with the largest number of participants was conducted by Kelly et al. [10]. The aim was to show levels of children’s cortisol in a school environment for a large sample of 15-year-old children (*N* = 2995, gender information missing) as well as examine differences in factors previously found to be associated with salivary cortisol measures. The participants were instructed to give the saliva samples (using Salivettes) around 5 min into completing a questionnaire (around 9 a.m. to capture the most rapid morning decline) and then again 30 min later. Participants who were not in school during the interview day (*N* = 137), failed to provide one or both saliva samples (*N* = 185), as well as 13 children who had extreme values (most likely due to contamination) were excluded from the analysis. The findings indicated gender differences where females had higher levels of cortisol at test 1 and a greater decline between test 1 and test 2 compared to males. In addition, other factors such as certain life events, sampling on Monday, time since awakening were also found to affect cortisol levels.

Secondly, Oskis et al. [40] measured the diurnal patterns of salivary cortisol in typically developing, healthy 9–18-year-old adolescent females (*N* = 61) over two consecutive weekdays. The cortisol was collected at home and at school depending on the timing of collection using passive drooling through a straw. The samples were collected at awakening, 15 min, 30 min, 45 min, 3 h, 6 h, 9 h, and 12 h post-awakening. Participants were instructed to not consume anything (apart from water), brush their teeth, or exercise in the 30 min prior to saliva sampling. The findings indicated that cortisol was highest 30–45 min after awakening and then declined over the day. The cortisol concentration pattern was relatively stable over the two sampling days for all participants. In addition, participants with a greater increase in cortisol after awakening exhibited greater daytime decline.

The studies discussed next in this review involve children with different disorders. Young, Sweeting, and West [43] investigated the level of morning cortisol and whether it was related to DSM-IV diagnosis and psychiatric symptoms. Fifteen-year-old children (*N* = 501) were asked to complete a computerized and DSM-IV compatible Diagnostic Interview Schedule for Children (DISC). Similar to Kelly et al. [10], saliva samples were collected 5 min and 30 min after participants started completing the questionnaire in school, approximately between 9 a.m. and 9.50 a.m. This research also found gender differences in the cortisol levels measured with only females with conduct disorder showing elevated morning cortisol. In addition to this, an interaction with mood disorder showed that females with more mood symptoms have higher morning cortisol while males with fewer symptoms have lower levels. No other associations between psychiatric symptoms and morning cortisol were found.

Two of the studies included children with Attention Deficit Hyperactivity Disorder (ADHD). Imeraj et al. [31] aimed to establish the diurnal profile through the day for 6–12-year-old children with ADHD (*N* = 33; *N* = 26 males, *N* = 7 females) and for comparison matched the children by gender and age to typically developing (*N* = 33) peers. Children provided 3 samples per day in school on 5 consecutive days. Findings indicated a cortisol difference where a subgroup of ADHD with Oppositional Defiant Disorder (ODD) had significantly higher morning and lower evening cortisol compared to the subgroup of ADHD. This also resulted in a steeper linear decrease in cortisol levels through the day.

Von Polier et al. [30] aimed to compare callous-unemotional (CU) personality traits, antisocial behavior, and comorbid ADHD symptoms with cortisol levels in 7–16-year-old male children and adolescents with early onset conduct disorder (EO-CD) (*N* = 37) and healthy matched controls (*N* = 28). Children provided 6 samples at school or hospital school and some at home, all with the help of study nurses that followed a protocol and kept a journal. The samples were provided immediately after awakening, 30 and 60 min later, at 12 p.m., 3.20 p.m., 3.40 p.m., and 7.30 p.m. The findings indicated no overall group differences, but a steeper decline of cortisol between the awakening and noon measurements for participants in the EO-CD group compared to healthy controls. Participants with EO-CD with higher levels of CU traits showed a significant lower level of cortisol in the first hour after awakening and to some extent throughout the day.

With a cohort of children with Autism Spectrum Disorder (ASD) Gabriels et al. [3] investigated diurnal cortisol in relation to repetitive behaviors in children. Male 3–9-year-old children (*N* = 21) were split into two groups: high repetitive behaviors (*N* = 11) and low repetitive behaviors (*N* = 10), using the caregiver-report Repetitive Behavior Scale Revised (RBS-R) and cortisol was collected four times per day (one of which was at school) on three consecutive days. The findings indicated that the children in the high repetitive behaviors group had significantly lower levels of cortisol compared to the children in the low repetitive behaviors group.

The last study in this subsection was conducted by Gustafsson et al. [9] who worked with 9–17-year-old children and teenagers with Obsessive-Compulsive Disorder (OCD) (*N* = 23; *N* = 10 males, *N* = 13 females) and compared them to a 6–12-year-old reference/control group (*N* = 240). This research has measured levels of cortisol as diurnal and acute measures, so only the relevant details of the diurnal cortisol collection will be discussed in this subsection as described above. The diurnal cortisol for the clinical group was collected on a weekend at home (at 8:30 a.m.; 10:30 a.m. and 9:00 p.m.) and was collected for the control group at the same time points but on a weekday at school and home. The findings indicated significantly higher cortisol levels for the early morning samples in the OCD group compared to the control group. When responding to a psychological stressor, the children in the control group showed a significant increase in cortisol levels, whereas the children in the OCD group showed a near-significant decrease. The difference in cortisol levels between the two groups was significant.

In terms of method of saliva collection, Gabriels et al. [3] is the only research discussed here to use color-coded Whatman filter paper to collect the saliva samples from the children. One study used passive drooling through a straw [40], whereas the rest of the studies in this section used Salivettes [9,10,31,43].

As most of the studies in this subsection have used Salivettes, there is some consistency in the collection method chosen, however, when considering the protocols across different research papers, the descriptions of procedural details vary. For instance, Imeraj et al. [31] instructed participants not to eat sour foods, drink, or brush teeth before the morning samples. They also asked adults to set alarms to remind children of collection during the day. This protocol appears to be robust as only 90 of the 1650 saliva samples (5%) were missing due to forgetting, deviating more than 15 min from the time, or insufficient saliva collection. Similarly, Gabriels et al. [3] also had an extensive collection protocol for saliva collection (no eating and brushing teeth prior to collection and no drinking 15 min before collection) as well as providing a research assistant for the children who would model the collection in order to convince the children to put paper in their mouths. Out of 21 children with ASD, only four children were unable to provide all the saliva samples due to non-compliance. As a result, they only provided 30 min after awakening and at 4 p.m. on two out of three days to show the overall decline. In addition, there were 15 random samples (some from all sampling times) missing from other children.

Although similar to the research discussed so far, von Polier et al. [30] have stipulated the rules of no brushing teeth or eating prior to collection of first 3 samples, they have also extended the criteria for their sample of children and teenagers up to the age of 16 to no smoking, drinking caffeinated or alcoholic drinks, and no exercise prior to collection (see [30], p. 3). To ensure correct sampling, a nurse was available to show the procedure and offer help, in addition to the written instructions provided. Participant compliance was monitored using a journal. In addition to limiting food, drink, brushing teeth, and exercise restrictions prior to collection of the samples, Oskis et al. [40] also sent text messages to the participants to remind them of the collection at the right time. For the first sample, the children had to reply with the time they woke up and the time of the collection of the sample. It appears that a text reminder is a successful method to ensure the collection of cortisol for typically developing children as there were no missing samples.

In this subsection, where the cortisol is used as a diurnal measure, the method of storage of saliva also differed depending on the project. It appeared that research articles had very little in common. Imeraj et al. [31] has been most specific when stating storage of saliva, stipulating that samples were centrifuged at 4 °C and then stored at −26 °C. Oskis et al. [40] on the other hand got the participants to freeze the sample as soon after collection as possible (for the school samples this was after the end of the school day) in their freezer at home, but the temperature was not specified. The samples were then transported using ice blocks and stored in the lab at −20 °C until further analysis. Furthermore, Kelly et al. [10] and von Polier et al. [30] both specified storing the samples straight after collection, but at the different temperatures of −20 °C and −60 °C, respectively. Expectedly, more differences were apparent for the research by Gabriels et al. [3], as the collection method used was different from all the other research discussed here. As a result, the processing and the storage were also different. The Whatman filter papers were separated by wax weigh paper to ensure the samples do not get contaminated. All collection times were color-coded to ensure that correct samples were taken at the set times and not mixed with different collection times. The samples were then stored in a booklet and allowed to air dry at room temperature. Although some may argue that the use of different techniques to collect saliva samples may influence the cortisol findings due to the different storage and processing, it has been shown that cortisol is highly stable, even if different storage methods were used [44].

### 3.2. Measuring Intervention Effects on Cortisol Levels

Six of the returned papers in this review focused on using salivary cortisol to measure the effects of different classroom interventions. Lupien et al. [20] evaluated a school-based program which taught adolescents about stress to establish whether it reduced students’ cortisol levels or depressive symptoms. Cortisol samples were obtained from 11–13-year-old adolescents (*N* = 504; *N* = 260 males, *N* = 244 females) who were transitioning to high school, which has been shown to be a stressful experience. The children attended two different schools with one school taking part in the intervention and the other one assigned as a control.

A much smaller sample was used by Butzer et al. [7] who collected saliva from 7–9-year-old children (*N* = 36; *N* = 20 males, *N* = 16 females) to investigate the effect of classroom-based yoga intervention on cortisol. A similar intervention, but based around mindfulness, was used by Schonert-Reichl et al. [6] and the effect it had on cortisol concentration was investigated. Saliva samples were obtained from children aged 9–11 years (*N* = 99) attending 4 different schools (1 class from each school). Similarly, Yoo, Lee, Lee, Shin, Park, Yoon, and Yu [42] provided a meditation program for 10-year-old children (*N* = 23; *N* = 13 males, *N* = 10 females) and took cortisol samples before and after the intervention, comparing them to a control group of the same age (*N* = 19; *N* = 7 males, *N* = 12 females). The children attended the same school but the two groups were in different classes.

A different intervention was used by Bunterm, Wattanathorn, Vangpoomyai, and Muchimapura [35] who investigated the effectiveness of different methods of teaching (Open Enquiry vs Conventional Learning) the same topic. Saliva was gathered from 10th grade Thai students (*N* = 86; *N* = 29 males, *N* = 57 females). As with the children who took part in Yoo et al.’s [42] study, all children attended the same school but were in two different classrooms.

Another study aiming to establish a difference in cortisol levels due to an intervention was conducted by Jager, Schmidt, Conzelmann, and Roebers [39], who used a physical activity and a cognitive engagement intervention. The children who took part were 6–8 years-old (*N* = 108; *N* = 59 females (54.8%)) and attended 9 different classes in the same region.

The intervention research presented has found different effects on cortisol levels of the children taking part. The findings of Lupien et al. [20] did not show an overall difference in cortisol levels before and after the 5-week interventions. However, adolescents who reported more anger prior to the intervention showed a larger decrease of cortisol levels while the children in the control group showed a slight but not significant increase in cortisol levels. In addition, females appeared to have higher levels of cortisol prior to the intervention only. Lupien et al. [20] conducted a multivariate analysis showing that only gender and anger were uniquely associated with cortisol intercept and the slope.

Butzer et al. [7] found that second graders showed a significant decrease in their cortisol levels after the 10-week intervention (30 min per week), but did not see an effect of a single yoga class.

The research projects on meditation and open enquiry interventions [35,42] found overall a significant reduction in cortisol levels due to the intervention between pre- and post-test.

In contrast, Schonert-Reichl et al. [6] did not find mean baseline cortisol differences between pre- and post-test after their 12-week (40–50 min per week plus 3 × 3 min mindfulness practice per day) intervention. However, differences in slope scores indicated little change for the children in the MindUP (mindfulness intervention) group, indicating no increase in stress, while the comparison children changed from a steeper to flatter diurnal pattern. However, the MindUP children had a significantly higher morning arrival cortisol at post-test compared to the BAU group and results are inconclusive.

Jager et al. [39] saw no significant pattern of cortisol change for the experimental group and the control group from pre- to post-test. When comparing the levels at post-test and follow up, a significant cortisol increase was found for the experimental group. Interestingly, for the experimental group, a subgroup showed increased levels of cortisol correlated with better inhibition abilities.

In terms of the methods of collection, some of the researchers chose to use the passive drool method [7,20,42], but others chose the cotton Salivettes [6,39], and one study did not specify which method of collection was used [35].

Several authors neglected to specify a detailed protocol which becomes apparent when reviewing the restrictions for participants prior to saliva samples. Some of the studies did not specify any restrictions (e.g., no food, exercise, or brushing teeth) prior to sampling [7,20,35,39], however, Lupien et al. [20] did emphasize that collecting two samples would potentially minimize confounding external variables such as food intake.

As these papers have not specified such details, it makes it difficult to replicate the research.

In contrast, other research provides more detailed protocols. Yoo et al. [42], for instance, stated that one hour prior to collection, participants were instructed not to consume any stimulating foods and 10 min before sampling children rinsed their mouth with cold water. A different protocol was followed by Schonert-Reichl et al. [6] where participants avoided food intake and physical activity at least 30 min prior to providing the sample. Furthermore, to ensure that children followed the protocol they were asked to write down their time of awakening and note the last food and/or liquid consumption before the cortisol samples and any medication taken during the day.

In addition to these differences, most of the research discussed so far in this section has not clearly stated the success level of adherence to the protocol. It is also unclear how many samples were not analyzed, and the non-compliance of children as well as the difficulties during the sampling are unknown. The exceptions were Schonert-Reichl et al. [6] and Bunterm et al. [35] who reported that the cortisol data had been reviewed to ensure there was a full set for all time points. Although the authors have not specified if there are any participants with missing data, one of the tables appears to indicate that all of the participants were included in the analysis.

The samples in this subsection have been collected pre- and post-intervention but the times of collection varied across the studies. Schonert-Reichl et al. [6] ensured the times of collection were the same for all participants (9 a.m., 11.30 a.m. and 2.30 p.m.). However, due to school preference, Lupien et al. [20] collected two samples at the beginning and end of each testing point (pre-, post- intervention, and follow up) at different times of day for the control school compared to the intervention group. The authors acknowledged that cortisol levels are higher in the morning and gradually decrease throughout the day, and this decline was accommodated for by using Latent Growth Curve Model (LGCM) during analysis. Yoo et al. [42], on the other hand, collected the samples from everyone pre- and post-intervention at the same time in the afternoon (2–4 p.m.) as there is a steady level of cortisol at that time, but it is unclear how many samples were provided by each child. Similarly, Bunterm et al. [35] also collected saliva samples before and after intervention in school, but they stated that only one sample was taken from each participant at each point and this was done between 7.30–8.00 a.m. for all participants. Jager et al. [39] also only collected one sample from each child before and after the testing and intervention as well as about 40 min later. This study did not specify the time of collection, as did Butzer et al. [7]. However, the time of collection for all participants was roughly the same. This was due to it being before and after their attention test of the first and last week, as well as immediately after the first and last yoga session.

From the studies presented above it is apparent that there is a large amount of variability in protocols used for salivary collection in schools. This variation appears to continue with regard to the storage of the saliva samples. With some studies storing the samples at −20 °C until analysis [20,39,42] while others shipped the samples for analysis immediately after collection [6], or have frozen the samples before shipment for analysis without stating the temperature they have been frozen and/or transported at [35]. Butzer et al. [7] did not specify how the samples were stored prior to analysis.

### 3.3. Baseline Measure

Four of the papers returned in the search [14,33,34,36] used the cortisol as a baseline measure and compared it to other measures. Lupien et al. [14] aimed to measure the levels of cortisol in children (*N* = 307) of different ages (6–16 years) from low (*N* = 167) and high (*N* = 140) socioeconomic status (SES) in order to establish any differences and their variation according to age.

The aim for the research by Alghadir et al. [33] was to investigate the effect of physical activity on depression in 7–18-year-old children (*N* = 150; *N* = 90 males, *N* = 60 female). A similar age group was used by Catherine et al. [36] who measured salivary cortisol in 9–12-year-old children (*N* = 89; *N* = 40 males, *N* = 49 females) to establish associations between cortisol, relationships, and behavior. The last paper in this section investigated the effects of stress and trauma on cortisol levels among 7–13-year-old school-aged children (*N* = 68, *N* = 30 males (44%), *N* = 38 females (56%)) [34].

These four research papers all found differences in the cortisol levels measured. Lupien et al. [14] showed that children in the low SES subgroup had significantly higher levels of cortisol compared to children from the high SES subgroup before the transition into high school. This was no longer significant once the children started attending high school.

Alghadir et al. [33] found that the reduction of scores of depressive symptoms correlated with lower levels of cortisol for physically active participants. In addition, the score of higher depressive symptoms in participants aged 12–18 years is linked to higher levels of cortisol.

Catherine et al. [36] detected a positive and significant association between afternoon cortisol and some of the other measures such as peer- and teacher-reported prosocial behaviors.

Bevans et al. [34] also found a difference for afternoon cortisol as it was influenced by the frequency of exposure to life stressors. Participants exposed to life stress within the last 12 months showed higher afternoon levels of cortisol. Furthermore, a combination of high levels of recent trauma and frequent exposure of trauma earlier in life related to lower morning, but higher afternoon cortisol. 

Regarding collection methods, Lupien et al. [14] chose filter papers as Salivettes rather than the cotton Salivettes used by most of the other research discussed so far. They collected two saliva samples in the morning at 8 a.m., before the neuropsychological assessment and 60 min later, at the end of the session. The children saw a demonstration from the principle investigator, showing how to provide the saliva sample before doing it themselves. However, it has not been specified whether the children had to follow any restrictions. The Salivette paper was then clipped on a drying device.

In Alghadir et al. [33] it is not clear which method of collection has been used to collect salivary cortisol or indeed how many saliva samples were taken from each participant. The authors did however specify that samples were collected between 9–11.30 a.m. following an overnight fast, but there was no mention of other restrictions such as brushing teeth or exercise.

Catherine et al. [36] used Salivettes, but in the cotton form to collect saliva samples at 9 a.m., 12 p.m., and 3 p.m. over four consecutive days. The participants recorded time of awakening and food intake prior to every sample collection, but it is not clear whether they had to follow any restrictions prior to providing the samples.

Like Alghadir et al. [39], Bevans et al. [34] also used cotton Salivettes to collect the saliva samples. The samples were collected at school between 7.45 a.m.–8.10 a.m. and 2.15 p.m.–3.00 p.m. Children had to rinse their mouth with water 15 min prior to sampling, but no detail has been provided with regards eating, drinking and exercise prior to collection.

When considering the storage of saliva samples, it is clear that different studies have used different protocols. Bevans et al. [34] kept the samples cool and then transported them to be stored at −70 °C until further analysis. In contrast, Catherine et al. [36] centrifuged the Salivettes within 3 h but did not specify how they were stored before analysis. Similarly, Alghadir et al. [33] have also centrifuged the samples straight away and then stored them at −80 °C until further analysis. As Lupien et al. [14] used a different method of collection, the protocol was also likely to be different. Information is given on storage, namely that the paper Salivettes were left on a drying device. No information on further handling and transportation for analysis is given.

None of the research discussed in this section specifies in detail whether the method and protocol of collection were successful. However, Catherine et al. [36] screened the cortisol samples for completeness at all time points. Furthermore, they specify that the method used is consistent with other published articles investigating the average daily patterns of HPA. In addition, Lupien et al. [14] also stated that this method of collection has been deemed as reliable in research with adults.

### 3.4. Cortisol as an Acute Measure

Nine of the papers that emerged from the literature search used salivary cortisol as a measure of acute stress [1,2,4,5,8,9,37,38,41].

Measures of cortisol were used in different contexts to answer different study aims. One study used cortisol as an acute measure after 12 min of intensive exercise for 9–10-year-old school children (*N* = 53; *N* = 27 males, *N* = 26 females) who were allocated into 2 groups, i.e., an exercise group (*N* = 32, *N* = 15 female, *N* = 17 male) and a control group (*N* = 21; *N* = 10 males, *N* = 11 females). Results showed no elevated cortisol levels after sports in the exercise group, but instead, lower cortisol levels in the control group who watched an enjoyable movie. This finding was mainly due to females in the control group [8].

Another study collecting salivary cortisol with children of a similar age (8–10 years) was conducted by Haines et al. [38], who investigated the effect of aircraft noise on stress responses, mental health, cognition, and cortisol. Child participants (*N* = 340; *N* = 170 males, *N* = 170 females) either attended school in a high-aircraft noise impact area (*N* = 169) or a low-aircraft noise impact area (*N* = 171). Haines et al. [38] did not report any significant effects for cortisol when comparing the children attending the high and low noise area schools.

Both Budde et al. [8] and Haines et al. [38] used Salivettes as the method to collect salivary cortisol. However, Budde et al. [8] specified that the saliva was collected around 11.30 a.m. to ensure the children had not eaten or drunk anything in the 2 h prior to sampling. The children then provided another sample after the 12 min of exercise (for the experimental condition) and 12 min of rest watching a film (for the control condition). Although in the research by Haines et al. [38], children also provided two morning samples, i.e., before and after testing, where the samples were 1 h apart and the time of collection has not been specified. The researchers screened for potential contamination of cortisol samples which could be caused by food, drink, medication, bleeding gums, physical activity, or stressful life events, rather than placing restrictions on participants. Only a subgroup of the participants provided saliva samples and the authors have not been explicit in stating if any of the samples have been discarded.

The remainder of the studies that will be presented in this section have been conducted with different clinical groups. Scherr et al. [41] compared boys with Fragile X Syndrome (FXS) and typically developing (TD) (*N* =105), using a verbal working memory assessment task. Boys were non-verbal mental age-matched and a subgroup of the total number of participants provided saliva samples (*N* = 80; *N* = 31 FXS; *N* = 49 TD). The baseline cortisol measures showed increasing levels of cortisol over time for boys with FXS, but differences between the two groups on baseline cortisol did not reach significance. Furthermore, boys with FXS had higher levels of baseline cortisol associated with poorer performance on the auditory memory task. FSX boys also had significantly higher reactant cortisol than the typically developing children in Year 1 of the study.

A further study was conducted with a different clinical group by Palma et al. [1] who investigated acute stress within the classroom in 6–17-year-old children and teenagers with ADHD (*N* = 38; *N* = 31 males, *N* = 7 females) and matched the sample by age, gender, and education with typically developing controls. The findings indicated that the control group had lower cortisol levels after the stressor compared to children with ADHD at time 1 and time 2 of sampling, which were taken 20 and 40 min after the stress test.

Then, Palma et al. [2] completed a follow up study, involving the comparison of 10–18-year-old children with ADHD (*N* = 37; *N* = 30 males, *N* = 7 females) to healthy controls (*N* = 22; *N* = 15 males, *N* = 7 females) on the factor of stress within the classroom. Unlike the first study, the follow up did not find any significant cortisol differences as a reaction to the stressor between groups, however, the ADHD group had a significantly higher baseline cortisol than the control group and significantly higher cortisol at baseline than at follow-up measures.

Working with boys with insecure/disorganized attachment, Beetz et al. [4] and Beetz et al. [5] investigated cortisol levels during a stressful task with different social support conditions with either a real dog, a toy dog, or a friendly person. The tasks were completed by 7–12-year-old and 7–11-year-old boys, respectively (*N* = 31/*N* =47). Both research projects found that children in the dog condition had significantly lower cortisol levels than the other support conditions during and after the stress test. Stroking the dog was also linked to lowered cortisol levels.

Details of the study by Gustafsson and colleagues [9] have been discussed earlier in the “Diurnal measure of cortisol” subsection as they also measured diurnal cortisol, but the results relevant for acute cortisol will be discussed here. As expected, the findings showed that the children in the OCD group had higher cortisol in the morning, which decreased through the day. When comparing the cortisol of the two participant groups, the children in the OCD group had significantly higher cortisol levels in the morning compared to the reference group. From the cortisol analysis in response to a stressor, the children in the reference group showed an increase in their response, while the children in the OCD group had a near significant decrease in cortisol. The difference in cortisol levels between the two groups as a reaction to a stressor was statistically significant.

The last study that will be discussed in this subsection was conducted by Fernald et al. [37] who investigated the relationship between growth and the response system in Nepalese children of typical and atypical physical height. Participants aged 8–10 years (*N* = 130; number per gender not specified) were divided into 2 groups: atypical physical height (*N* = 66) and typical physical height controls (*N* = 64). There was only a significant difference with atypical physical height children having blunted physiological response to stress when taking into account both cortisol measure and heart rate.

The research shown here has mostly used cotton Salivettes [2,4,5,8,9,37,38,41] to collect the saliva samples, with one study using both passive drool and Salivettes [1]. However, when discussing the protocol of collection, again, variation across studies becomes apparent. Scherr et al. [41] collected saliva samples 15 min before the assessment as a baseline measure (9 a.m.) and straight after the assessment as a measure of the effect of the assessment (12 p.m.), where 95% of participants took part in school. The children were encouraged to be well rested and if unwell, parents were asked to reschedule the assessment, although this would have been difficult to ensure. In addition, the participants confirmed that they have not consumed any foods or liquids 30 min prior to saliva sampling. Similarly, Budde et al. [8] also took a sample before and after the activity, specifying that the first sample was at 11:30 a.m., which ensured that children have not eaten in the last 2 h. Another study, which only took two samples, pre- and post-activity, was conducted by Haines et al. [38]. Saliva was collected in the morning before the cognitive test and 1 h later after testing. The samples were screened for contamination from food, drink, and recent life events but there do not appear to be restrictions placed on the participants prior to providing the sample. Similarly, Gustafsson et al. [9] also collected two samples, one before and another 30 min after the stressor. However, the paper does not specify the times of saliva collection for the acute measure.

Considerably more samples were taken by Palma et al. [1] and Palma et al. [2] who collected the saliva samples at the same time points: before the stressor (Continuous Performance Test (CPT)) and then 20 min, 40 min, and 60 min after the CPT was completed. The same protocol of no food or drink intake 30 min prior to collection and mouth rinsing using filtered water was also followed in both studies. Beetz et al. [4,5] also collected samples at more time points before, during, and after the stressor occurred. They have a detailed protocol in which they show when exactly the 5 samples were gathered ([4], page 356). Similarly, Fernald et al. [37] collected saliva samples at 5 points during the psychological testing (upon arrival, after completion of digit span task, 10, 20, 30 min after completion of digit span task). A further 6th sample was collected on waking the day after testing took place. Although the study by Fernald et al. [37] also did not specify whether there were any restrictions for participants prior to them providing a saliva sample, children were given a snack and a drink after the first saliva sample to ensure they were not hungry during the psychological testing. This approach raises the question of whether the following saliva sample (taken about 20 min after) was affected by the snack.

In addition to the already emphasized variation from one study to the next, some have failed to mention any restrictions, such as but not limited to, no food or drink prior to sampling and no vigorous exercise [4,5,9].

When considering the quality and usefulness of protocol it is important to view the success rate of collection. Missing cortisol data can lead to exclusion of participants with similar characteristics (e.g., behaviorally challenging/non-compliant children) or family background (e.g., children from low SES background) which may in turn result in inaccurate conclusions which are not suitable to be generalized. Palma et al. [1] stated that three participants were excluded due to missing data, although it is unclear whether the missing data was cortisol samples, whereas Gustafsson et al. [9] indicated that 240 of 336 reference group children (participants used from another study conducted by the same research team) provided the acute measure of cortisol and one child in the OCD group did not provide both saliva samples. Similarly, research by Scherr et al. [41] has established that some samples were missing at random due to non-compliance, excessive artefacts or evaporation of samples, but it is not clear how many samples were missing. However, they carried out checks and confirmed that participants were still matched according to mental age. In addition, the research by Palma et al. [2], and Haines et al. [38] did not state whether any children were excluded, although Haines et al. [38] presented statistics of child, parent, and teacher responses, which would suggest no children were excluded but some had missing data. This makes it difficult to draw conclusions about the usefulness of the methods used and their advantages and disadvantages. Finally, Fernald et al. [37] confirmed that complete cortisol results were available for 127 out of 128 children (99%).

In terms of storage of the samples, again there was a wide variety of approaches in the studies included in this section. Palma et al. [1], Palma et al. [2], Scherr et al. [41], and Haines et al. [38] did not specify how the samples were stored before analysis or how quickly they were analyzed after collection. In comparison, Beetz et al. [4] and Beetz et al. [5] froze the samples at −20 °C until later analysis, whereas, Gustafsson et al. [9] mailed the samples to the laboratory to be centrifuged up to 5 days after the samples were first collected. Then they were stored at −20 °C for 6 months before analysis. A different approach was taken by Fernald et al. [37] who centrifuged the samples briefly, then transferred samples into cryovials and froze them at −20 °C and transported them from Nepal to the UK for analysis using ice packs to keep them cool. It is, however, unclear how long it took for the samples to arrive. Budde et al. [8] on the other hand did not specify either the storage or the analysis of samples. These differences show once more the lack of a gold standard protocol once the saliva was obtained.

## 4. Discussion

This review aimed to evaluate the feasibility of cortisol collection in schools for future research and to make recommendations for best practices. Twenty-five research studies were found to fulfil the criteria for the systematic review, all collecting salivary cortisol from school-aged children from within the school environment. Comparison of these studies found large variation between the studies. In addition to the broad range of aims and results, the articles in this review also show a wide variability in populations and protocols used, in approaches to sampling and storage, and the level of methodological detail provided.

For instance, most of the studies included in this review who have had participants of both genders report the number of males and females taking part [1,2,3,6,7,8,9,30,31,33,34,35,36,37,38,39,42]. However, others failed to report this information [10,14,43] despite including gender in their statistical analysis and reporting it within their results section.

Design variability and limitations also require consideration. Some of the research reviewed here did not need to use a control group due to their aims of the study. For example, some research examined differences between repetitive behaviors of children with Autism [3], levels of cortisol in typically developing children in a naturalistic school environment [10], and looking at the HPA axis functioning in children with psychiatric disorders [43]. However, some of the research that did not include a control group would have benefitted from its inclusion. For example, Butzer et al. [7] administered a yoga classroom intervention, which included all participating children in the intervention and as a result did not have a control group to compare their yoga group against. This raises questions with regards to the findings and whether the reduction of cortisol over time was due to the yoga itself or other external factors, which the whole school may have been exposed to such as a new head teacher. Similarly, Beetz et al. [4] and Beetz et al. [5] have 3 social support conditions for children with insecure attachment to establish which is the best support in a stressful situation. Although Beetz et al. [4] stated that the toy dog condition is a control group and Beetz et al. [5] report that the toy dog and the person condition are control groups, strictly speaking they are other types of support. A strict control would be if children undertook the stress test on their own, without any social support. The inability to compare the results to a strict control group in this type of research may pose questions about how effective the support of the toy dog and human condition is and how that would compare if the child was to complete the task without any social support. Although the levels of cortisol for the children in the dog condition are significantly lowered, would this still be the case if compared to a strict control? Perhaps it could be argued that the demand placed by the human (although a friendly student, children may find it difficult to communicate and relate to her) or the disappointment of not being with the real dog is the reason for the difference in the levels of cortisol rather than the dog itself. In contrast, some papers have included a strict control [1,2,6,20,30,31] and some have even matched participants by factors such as non-verbal mental ability, age and gender, when comparing children with different profiles [1,2,30,31,41].

There are further differences when discussing the control groups, as some of the studies in this review chose to allocate the children taking part into conditions randomly [8] while others chose to have the control groups in different schools to their intervention groups [6,20]. In doing so, children would not be upset for not taking part in the intervention, or anticipate their turn to take part, but this design does not eliminate across school variation. It can therefore be argued that any differences or improvements in cortisol and experimental results could be due to other factors related to the differences in school settings, not necessarily the intervention.

Despite this criticism, it is important to point out that when conducting research within a school environment there are a lot more factors to consider compared to lab-based research. For instance, Lupien et al. [20] allocate children into different groups so that each participating school only took part in one condition to ensure that the control children do not learn about the programme and apply it themselves. However, this design is flawed as both schools start at a different time during the day. As a result, children would be waking up at different times, which could affect the level of cortisol secretion and impact on results. In addition, the fact that children in the intervention school were able to take part at any time of day, compared to children in the control group who could only take part in the afternoon would mean that cortisol concentrations between the two groups would clearly vary as cortisol declines steadily through the day. The authors have controlled for the levels of cortisol to overcome this issue, by using a Latent Growth Curve Model, which estimates separately but simultaneously the level of cortisol concentration in children before and after the intervention. However, having a lower level of cortisol to begin with would mean that it would be more difficult to detect a further decrease as the reduction will not be as prominent. Despite this not being an ideal design, this shows the complexity of working within schools where the taught lessons, children’s activities, and length of school day have to be taken into account.

The complexity of research within applied settings emphasizes the need for more research to be tailored towards these settings to enable the obstacles to be overcome in order to find more realistic results, ensuring that findings are more easily generalized across settings. In addition to this, the aim of the study required schools to be situated in different areas such as when comparing children exposed to living in an area of high and low aircraft noise [38], so across-school variability was inevitable. Some studies limit this design issue by including many different schools to take part in both the control and experimental groups [37], whereas other researchers have minimized school differences by ensuring that all the schools taking part are from the same School Board Commission, which ensures similar schooling procedure. Another attempt to find a solution to this issue was employed by Yoo et al. [42] who used the same school but the children were part of different classrooms that were located away from each other.

Differences between research projects continue when reviewing the research with children with learning difficulties and other mental health diagnosis, especially when considering the recruitment and selection using the diagnosis of the child. Although most of the research has used DSM versions for diagnosis [1,2,3,9,30] one study has confirmed the previous diagnosis using the Autism Diagnostic Observation Schedule along with parent questionnaires [3], two studies have used the SNAP-IV (A version of the Swanson, Nolan, and Pelham Teacher and Parent Rating Scale, based on the DSM symptoms) to establish a diagnosis of ADHD, as it is standardized for Brazil [1,2], and another study has only used the previous diagnosis, but further analyzed the psychopathic traits using Antisocial Personality Screening Device [30]. In addition to these differences, two studies have not specified the diagnosis process the children have undergone and which method of diagnosis was used [31,41]. This indicated no clear selection criteria when conducting research with different clinical groups. Being clear and consistent with the inclusion criteria is particularly important as using biological measures such as cortisol can provide us with more in-depth information about the differences between children with difficulties and typically developing children. This is particularly important as children with different diagnoses have different levels of cortisol [12] indicating that there are physiological differences for children who show varied deficits. As children have such differences, it would suggest that the areas targeted to help children with different deficits are not the same. Finding out more specific underlying information about children with different disorders and deficits could lead to better focused, more specific support. It is vital that we can accurately generalize findings to specific groups of children.

In turn, the same selection criteria should ideally be used to ensure enough data is collected for children who show similar deficits even if diagnosed with the same disability. If research does not narrow down the groups enough to make them more specific, the help provided may not be offered to the child that would benefit most and therefore may not be deemed appropriate. For instance, recent research has found that children with low functioning autism have got higher cortisol levels compared to children with high functioning autism and typically developing children [45]. These results have indicated that different strategies would be needed for children with low and high functioning ASD, perhaps due to their anxiety levels. This emphasizes the importance for strict inclusion and exclusion criteria in research with children with different diagnosis in order to discover accurate findings about subgroups of children who have the same diagnosis but experience different difficulties.

In addition to this overall lack of standard procedure and lack of detailed and published protocols to enable replication, success rates of obtaining the samples from children were also often not reported. This in turn results in little knowledge about non-compliance of children [1,4,10] and problems within protocols for collection. From all the research discussed in this review only a handful of papers reported this information. For instance, Young et al. [43], stated that 93% of the total participant subsample provided valid cortisol samples and completed the questionnaire provided. Fernald [37] also provided this data. This was very similar to the success rate reported in the study by Gabriels et al. [3], where 95% of children (1560 out of 1650) were able to provide all saliva samples. In contrast, other papers have stated that they have used protocols from previous research rather than explicitly changing their own protocol [36]. As so few of the papers report the success rate of obtaining samples that are suitable for analysis, it is difficult to make an informed decision on the protocols and the most successful guidelines to use.

## 5. Recommendations for Future Research

Through the research discussed here, it is clear that salivary cortisol is a physiological marker that has been successfully measured across a variety of research in school-based settings. Cortisol has been used to measure the effect of interventions, the response to stressful events, in addition to its use as a one-off comparison measure. Such flexibility, and the fact the collection is not an intrusive process, encourages its use in applied settings such as schools. In addition to the flexibility, measuring the level of cortisol gives an insight into the physiological changes that occur within the participants at a given time. It is feasible to collect and fairly stable [44], which makes it a suitable choice for research conducted in applied settings such as schools. Being a fairly stable substance, it also enables it to be transported and analyzed by a third party. In addition to this, the different methods of collection of salivary cortisol (e.g., Salivettes, passive drool) ensure that saliva can be collected from young children and children with special needs, further widening its usefulness within research.

However, by discussing the research on the collection of cortisol in schools it is apparent that the collection guidelines and protocols vary across studies. This can result in different findings and potentially contaminated samples depending on the guidelines participants have been given. For example, it is well established that intake of food and exercise [46] among other factors can affect cortisol. In addition to this, the instructions provided in different studies have also varied from some researchers training children beforehand to provide the sample, to others only providing an instruction booklet. Furthermore, some researchers have chosen to monitor compliance using diaries while others have reminded participants of sample collection via text message or an alarm. One thing that is clear is that none of the studies were transparent with the whole process of the saliva collection and detailed protocol as well as reporting items like the success rate in obtaining the samples. This is particularly crucial as much of the research was also conducted with children with different disorders, some who have more than one diagnosis. These participants are likely to have different levels of cortisol depending on the comorbidity of their disorders, which makes it even more difficult to generalize findings and form conclusions.

We recommend that future research provides full details on the methodological choices, protocol of collection and storage, and success rate of obtaining salivary cortisol samples in schools and other applied settings. Being more transparent will enable the establishment of a gold standard within the field. This would further inform research and ensure better, more consistent practice, leading to more robust and more comparable findings.

Our first recommendation for future research, based on the research evaluated so far, is that both Salivettes and passive drool seem suitable for saliva collection with school children. Only one research article [23] recommended to use either passive drool or sorbettes, as their research comparing collection methods showed that “the Salivette yielded unstable and variable concentrations” (p. 560). They also found the sorbette in comparison to the Salivette to be safer to use, especially for children with ASD as they are securely molded to a handle (p. 570), however, most other research used Salivettes or passive drool.

The second recommendation is that the protocol for collection is explicit and detailed, to ensure comparable and replicable collection methods within and between studies and to avoid contamination. It should include instructions for participants and depending on the participants recruited, a training day or modelling of sampling method. Instructions should also include restrictions of no food, drink, vigorous exercise, brushing teeth 30 min to 1 h prior to sampling if possible. Recent negative life events should also be recorded if possible. It should be ensured participants are in good health and any medication taken currently is recorded.

In terms of the procedure of cortisol sampling we suggest that it is best if researchers are present during collection to ensure consistency of data collection, or, if this is not possible, a strict time for collection must be set and a diary to monitor adherence to collection time set. If this is not possible a text message to remind participants of the collection time approaching is likely to be as successful.

Finally, it is vital for the success rate of saliva collection to be reported clearly along with the protocol as this will enable researchers to establish the most useful and least time-consuming method of collection. Recommendations for best collection practice are depicted in overview in Table 3 below. Overall, as many factors can influence saliva cortisol levels, consistency and transparency of the methods of collection and storage are key to obtain reliable and comparable data.

In addition to these recommendations on collection, protocol, and storage, we also have recommendations for the research design and reporting of the projects. To enable future research to draw accurate conclusions and make comparisons, the design needs to have an appropriate control group, with participant numbers for sufficiently powered data analysis. Detailed demographics (e.g., including exact age and participant gender and, if possible, SES), should also be explicitly reported to inform the field, especially as, much of the research has not reported the findings in relation to gender differences and factors such as SES.

Lastly, if the research involves participants with special needs, investigators should attempt to collect background information as well as record deficits the participants have rather than just the diagnosis. This will enable the cortisol findings to be related to a more specific group of participants as many people suffering from the same disorder often struggle with different deficits.

Following these recommendations and improving on the research conducted so far will ensure that future research is clear, replicable, and concise, with strong scientific rigor. The results will be generalizable and further research will be enabled to fill the knowledge gaps.

## Figures and Tables

**Table 1:** Quantity and quality of research articles according to Oxford Centre for Evidence-Based Medicine 2011 Levels of Evidence as used in this review.

Level	Levels of Evidence	Quantity of Articles
1	Systematic review of randomized trials or n-of-1 trials	0
2	Randomized trial or observational study with dramatic effect	9
3	Non-randomized controlled cohort/follow-up study	16
4	Case-series, case-control studies, or historically controlled studies	0
5	Mechanism based reasoning	0

**Table 2:** Overview of results.

First Author Year	Participants					Salivary Cortisol Collection					
	N	Male (% Only Where Stated)	Female (% Only Where Stated)	Age	Cohort Details	Control Group	Cortisol Measure	Method	Protocol	Time	Storage
Alghadir 2016 [33]	150	90	60	7–18 years	Typically Developing	N/A	Baseline comparison with other measures	Not known	Saliva collection after overnight fast	Morning: 9.00–11.30	−80 °C
Beetz 2011 [4]	31	31	0	7–12 years	Insecure/Disorganised Attachment	No	Acute	Salivette	Not known—researcher present when saliva sampling	Pre stress test, 7 min, 20 min, and 13 min later, post test	−20 °C
Beetz 2012 [5]	47	47	0	7–11 years	Insecure/Disorganised Attachment	No	Acute	Salivette	Not known—researcher present when saliva sampling	Pre-stress test, 7 min, 20 min, and 13 min later, post test	−20 °C
Bevans 2008 [34]	68	40%	60%	7–13 years	Typically Developing	N/A	Baseline comparison against measures	Salivette	Mouth rinsed with water 15 min prior to sampling	1 a.m. sample 7.45–8.10 1 p.m. sample 2.15–3 p.m.	Stored in cooler, then university freezer −70 °C
Budde 2010 [8]	53	27	26	9–10 years	Typically Developing	Yes	Acute	Salivette	First sample at 11.30 No food in last 2 h. Second sample approx. 12 p.m.	Pre- and post- exercise/movie watching	−20 °C
Bunterm 2012 [35]	86	29	57	10th grade Thailand	Typically Developing	Yes	Intervention	Not known	Not known	Pre and post intervention 1 sample pre and post between 7.30–8.00 a.m.	Frozen and sent for lab analysis
Butzer 2015 [7]	36	20	16	2nd and 3rd grade	Typically Developing	Yes	Intervention	Passive drool	Instructions given prior to sampling	Pre- and post-stress test in week 1 and 10, after yoga session	Frozen, temp. not specified
Catherine 2012 [36]	89	40	49	9–12 years	Typically Developing	N/A	Baseline Comparison against measures	Salivette	Recorded time of awakening and recent food intake before every sample	9 a.m., 12 p.m. and 3 p.m. on 4 days	Samples centrifuged up to 3 h after collection
Fernald 2003 [37]	130	Not stated	Not stated	8–10 years	Children with delayed physical growth and typically developing children	Yes	Acute	Salivette	Not known	Arrival, after digit span task, 10, 20,30 min after digit span task. 6th sample after school	Centrifuged, then stored at −20 °C
Gabriels 2013 [3]	21	21	0	3–9 years	ASD	N/A	Diurnal	Color-coded Whatman filter paper	No eating, brushing teeth before collection, no drinking 15 min before collection. Collection demonstrated by RA.	Awakening, 30 min later, before lunch, 4 p.m. on three consecutive days	Stored in booklet and dried at room temperature
Gustafsson 2007 [9]	23 + 336	10	13	9–17 years	OCD Typically Developing	Reference group—previous study	Acute	Salivette	Not known	Before and 30 min after stressor—time of day not specified	Centrifuged, then stored at −20 °C for 6 months
Haines 2001 [38]	340	50%	50%	8–10 years	Typically Developing	N/A	Acute—measuring cognitive performance	Salivette	Screening: touching Salivette, food, medication, life-events, smoking, physical activity, mouth infection.	Morning	Not specified
Imeraj 2012 [31]	66	52	14	6–12 years	ADHD Typically Developing	Yes	Diurnal	Salivette	No sour food, drink or brushing teeth 30 min prior to sampling	After awakening, 30 min later, noon, 4 p.m., 8 p.m.	Home keeping during collection. Then collected, centrifuged stored at −20 °C till analysis
Jager 2014 [39]	104	45.2%	54.8%	6–8 years	Typically Developing	Yes	Acute—measuring intervention	Salivette	Not specified	School morning	−20 °C
Kelly 2008 [10]	2995	Not stated	Not stated	15 years	Typically Developing	N/A	Diurnal	Salivette	Not known—researcher told teenagers when to put Salivette in mouth	Five min after start of questionnaire, 30 min later, about five min before end of session	−20 °C
Lupien 2001 [14]	307	Not stated	Not stated	6–16 years	Typically Developing	N/A	Baseline comparison against measures	Salivette—filter paper	Instructions provided before sampling, demonstration of collection	Two samples pre and post assessment. Approx. 8 a.m., 9 a.m.	Paper clipped to drying device
Lupien 2013 [20]	504	260	244	11–13 years	Typically Developing	Yes	Intervention	Passive drool	Provided with oral instructions	One sample at beginning and one at end of each session. Pre-intervention, post-intervention and at follow-up points	−20 °C
Oskis 2009 [40]	61	0	61	9–18 years	Typically Developing	N/A	Diurnal	Passive drool straw	Participant information pack explaining collection. Only consuming water 30 min before sample, no exercise or brushing teeth.	CAR: awakening, 15, 30, 45 min after. 3, 6, 9, 12 h after awakening. On 2 school days.	−20 °C, home. Frozen straight away or after school.
Palma 2012 [1]	76	62	14	8.7 years mean age	ADHD, Typically Developing	Yes	Acute	Passive drool, Salivette	Rinsing with filtered water prior sampling. No food/drink 30 min prior to sampling	15 min before stress test, 20, 40, 60 min post stress test	Not known
Palma 2015 [2]	59	45	14	10–18 years	ADHD Typically Developing	Yes	Acute	Salivette	Rinsing with filtered water prior sampling No food or drink 30 min prior to sampling	15 min before stress test, 20, 40, 60 min post stress test	Not known
Scherr 2016 [41]	105	105	0	7–13 years	Typically Developing and Fragile X syndrome	Yes	Baseline—first assessment Acute—final assessment	Salivette	No food or drink 30 min prior to saliva samples.	Pre and post assessment Time not specified	Not specified
Schonert---Reichl 2015 [6]	99	Not stated	Not stated	9–11 years	Typically Developing	No	Intervention	Salivette	No food and physical activity min 30 min before sample collection. Researchers assisted collection. Children recorded time of awakening.	9 a.m., 11.30 a.m. 2.30 p.m.	Shipped analysis after collection
von Polier 2013 [30]	75	75	0	7–16 years	EO-CD CU traits Typically Developing	Yes	Diurnal	Salivette	Oral and written instructions for adults. Rinsed mouth pre- sampling. No smoking, eating, drinking caffeinated or alcoholic drinks, vigorous exercise or brushing teeth pre- first three samples. Journal for compliance.	Awakening, 30, 60 min after, 12 p.m.,15.20, 15.40, 19.00	Frozen imme-diately −60 °C
Yoo 2016 [42]	42	20	22	10 years mean age	Typically Developing	Yes	Measuring intervention effect	Passive drool	No stimulating food consumption 1 h before sampling. Rinsing mouth 10 min before collection.	School day 2–4 p.m.	−20 °C

**Table 3:** Recommendations for best practice in cortisol collection with school-aged children.

Factor to Consider	Recommendation for Best Practice
Method of collection	Salivettes and passive drool appear to yield similar results and are equally popular in research with children. For greater consistency and higher data reliability, we advise not to mix the methods of collection.
Protocol for collection	Protocol must be explicit, detailed and clear to enable replication.
Guidelines prior to collection	The following guidelines should be followed where possible: Food/drink—no food or drink 30–60 min prior to collection, except water. Exercise—no vigorous exercise 30–60 min prior to collection. Brushing teeth—no less than 30–60 min prior to collection.
Collection training and compliance	Researcher to train children and parents/teaching staff on do’s and don’ts when collecting cortisol based on the collection method chosen. Where possible and necessary, researchers to demonstrate how to collect saliva sample. Where possible and necessary, a practice session to be arranged prior to sampling.
Collection	Researchers to be present where suitable. If researchers cannot be present: Adults to have training and knowledge of protocol. Provide additional take-home instructions for both adults and children (written form or instructional video). Diary to be provided to monitor adherence to collection times. A call or text message to be sent to adult to remind of sample collection. If possible, use of automated reminder tools activated by adults or children on awakening for CAR measure (e.g., mobile phone).
Timing	Timing of sample collection must be respected as follows: Cortisol Awakening Response: collect saliva samples at 0, 30, 45 min post-awakening (see Stalder et al. [47] for details). Baseline measure: collect saliva samples at the same time every day, within 30 min interval, and ideally on 3 separate days. Acute/intervention measure (up to 1 h duration): collect saliva sample within 30 min pre-stimulus, and 25–30 min based on the peak of the intervention. If possible, control saliva samples should be taken the previous day at the same time or baseline to assess whether variations in cortisol levels are due to normal diurnal variations or to the stimulus/intervention. Additional samples could be collected at regular intervals (every 25–30 min) after the stimulus ended to assess time lag for the cortisol levels to return to the pre-stimulus cortisol levels.
Factors to record	Participants or parents/guardians to record the following through questionnaires or a diary where possible: Life events which may affect cortisol (e.g., parent divorce, moving school). Medication taken currently. Events in last 24 h.
Storage	Different storage procedures are possible, however the same procedure should be strictly followed throughout the entire study, including the storage timing in each condition (order from most to least recommended): Freezing samples immediately at −20 °C if possible. Place samples immediately in cold box, dry ice or fridge maintained at relative constant temperature (+4 °C recommended) up to 7 days, then freeze samples at −20 °C. Less ideal, but possible if necessary: Samples kept at room temperature up to 4 days, then freeze samples at −20 °C. Samples kept at room temperature up to 4 weeks is not recommended, after 4 weeks at room temperature samples are not usable. Long term storage (>12 months), −80 °C storage is possible. Avoid freeze/thawing saliva samples if possible, and not more than 4 times, and follow the same procedure for all samples. For Salivette samples, an extraction procedure is recommended before freezing the samples.
Reporting outcome	Researchers must be transparent about the participant group characteristics and the cortisol collection procedure. The following must be stated clearly: Protocol of saliva collection and storage. Success of collection protocol (i.e., how many samples were collected, how many samples did not have sufficient saliva for analysis or were contaminated). Information on where analysis was conducted and analysis details (ELISA serial number, inter- and intra-variation coefficient, assay sensitivity, dilution factor). We recommend that all study samples are analysed in the same laboratory.
